# Posttraumatic carotid-cavernous fistula: Pathogenetic mechanisms, diagnostic management and proper treatment. A case report

**DOI:** 10.4317/jced.52913

**Published:** 2016-04-01

**Authors:** Ana-Belén Marín-Fernández, Paolo Cariati, María Román-Ramos, José Fernandez-Solis, Ildefonso Martínez-Lara

**Affiliations:** 1 Maxillofacial Surgeon. Hospital Universitario Virgen de las nieves, Granada, Spain; 2 Oral and Maxillofacial surgery resident. Hospital Universitario Virgen de las nieves, Granada, Spain

## Abstract

Carotid-cavernous fistulas are an uncommon diseases characterized by abnormal communications between arteries and veins located in the cavernous sinus. According with Barrow´s classification they could be divided in two groups: direct and indirect. The typical symptoms showed by theses pathologies are: pulsating exophthalmos and orbital blow. The present study describes a case of direct posttraumatic carotid-cavernous fistula in a 26 years old man. Furthermore, we present the images that we used to make the diagnosis. In this light, we decided to treat this case with endovascular approach after considering several therapeutic options. The aim of the present report is twofold. First, we examine the importance of the proper management of the direct posttraumatic carotid-cavernous fistula. Second, we describe this rare syndrome with the goal of proposing suitable treatments.

** Key words:**Carotid cavernous fistulas, pulsating exophthalmos, orbital blow, endovascular approach, Barrow´s classification.

## Introduction

Carotid cavernous fistulas (CCF) are abnormal communications between the carotid artery and the cavernous sinus (1). Thus, these condition provoke a pathological arteriovenous shunt. According with Barrow´s classification, CCF could be classified in two groups: A) direct and B) indirect (1). Specifically, direct CCF usually present high flow, whereas indirect CCF typically show low flow. Moreover, these entities present different etiology. In fact, direct CCF are usually caused by severe head injuries or rupture of cavernous aneurysms. In addition, they have also been linked with other surgical trauma such as rhinoplasty, orbital floor fractures reductions, partial maxillectomy, nasopharyngeal biopsy and Le Fort 1 osteotomy (2). In contrast, the etiology of most indirect CCF is idiopathic. Interestingly, the symptoms of CCF can vary widely. Indeed, the symptoms included chemosis, conjunctival bleeding, diplopia, eyelid swelling, intense proptosis and vision loss. More deeply, the severity of each case is deter-mined by venous return capacity and by quantity and speed of the blood flow. 

As regards the diagnosis, angiographic study is mandatory. In this line, several paper reported that this procedure is essential for the proper management of CCF. In fact, angiographic study not only allows the correct classification of each case but also determine the treatment strategy. Indeed, direct CCF are usually treated with endovascular procedures (3-5). In contrast, the treatment of indirect CCF is more controversial. In this context, is important to stress that most of indirect CCF resolve spontaneously. Thus, several cases of indirect CCF only requires a clinical monitoring.

## Case Report

We describe the case of a 26-year-old man who suffered a severe cranio-facial trauma in 2004, caused by a car accident. Consequently, the patient presented subarachnoid hemorrhage and panfacial fracture. After an initial assessment in the emergency services, the patient was admitted to the intensive care unit of our Hospital. Here, the profesionals involved in the case decided that the subarachnoid hemorrhage did not require surgical treatment. Notwithstanding the above, the patient needed close monitoring in the intensive care unit for several week. Therefore, in the light of these developments, we also adopted a conservative attitude with regard to the facial trauma. In fact, during ICU stay the patient presented an unstable neurological status. Due to all this, we decided to reassess the patient in a year with the aim to estimate the fallout of trauma. Finally, when the patient came to the outpatient department of Maxilofacial unit we could observe the typical signs of CCF. Indeed, the physical examination of the patient showed zygomatic-orbital dystopia with pulsating exophthalmos and chemosis of the right eye. Moreover, the patient referred decreased vision in this eye. In addition to all this, we note the presence of an orbital murmur. This finding made us suspect the existence of CCF secondary to trauma. Bearing this in mind, a craniofacial CT scan was performed in order to confirm the diagnosis. As expected, this test showed the presence of CCF draining into the right superior ophthalmic vein. Thus, we decided to present the case to the interventional neuroradiology service of our Hospital.

**Figure 1:**
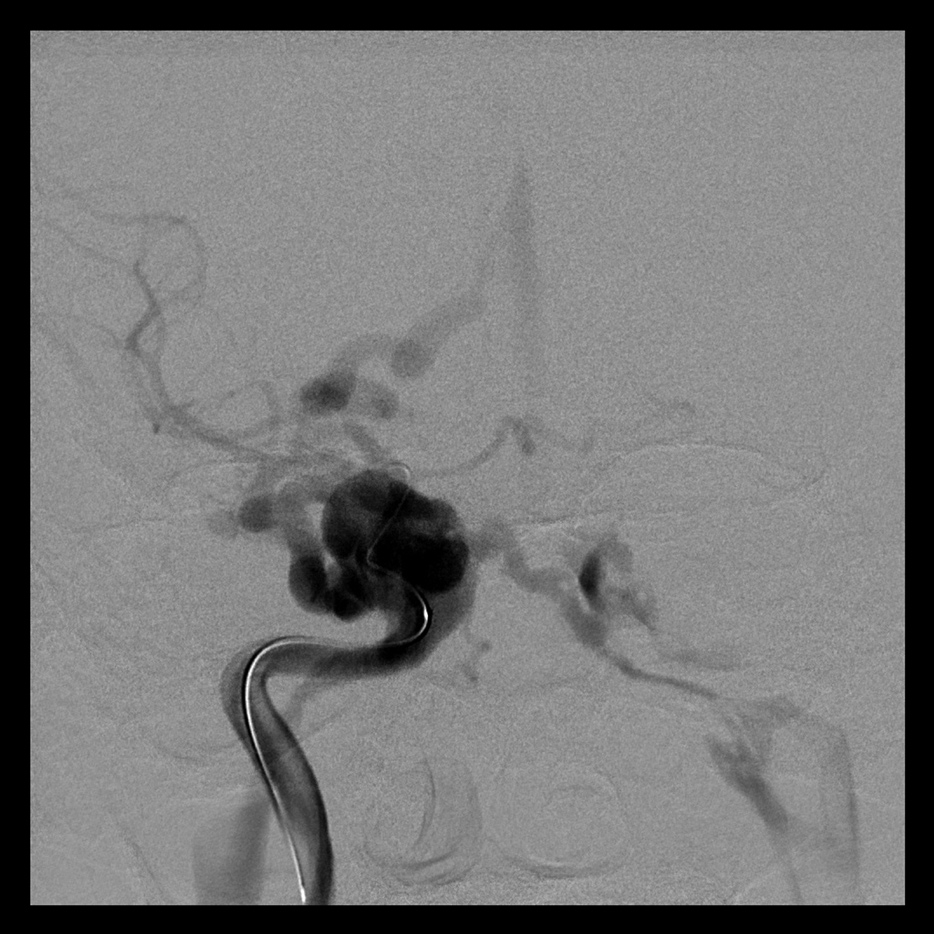
AP head arteriography. CCF before treatment with endovascular approach.

**Figure 2:**
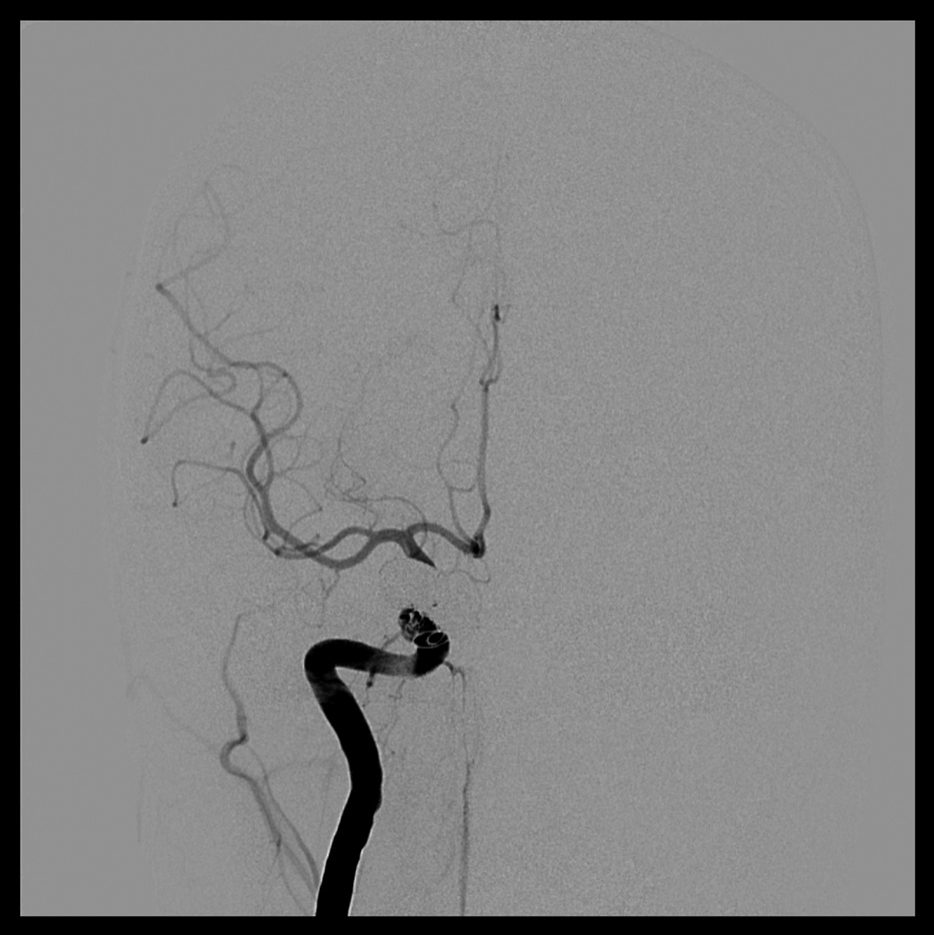
AP head arteriography. CCF after treatment with endovascular approach.

**Figure 3:**
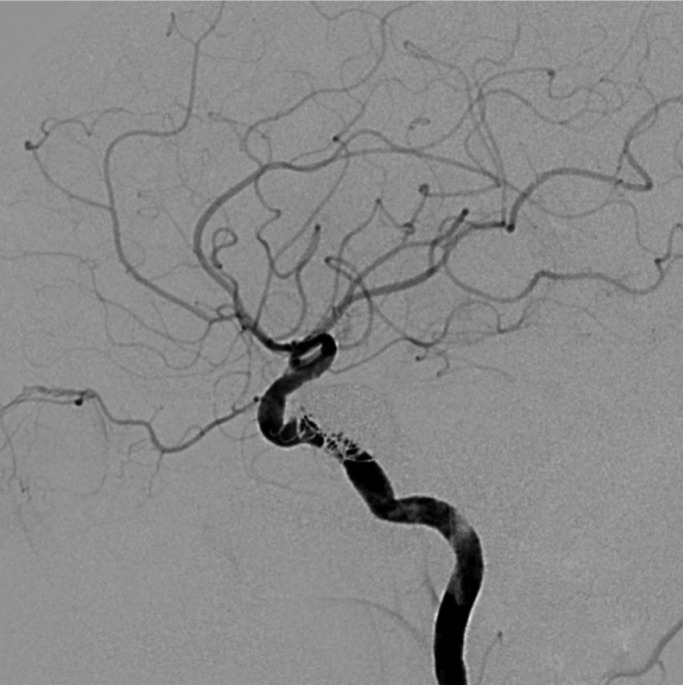
LL head arteriography. CCF after treatment with endovascular approach.

After evaluating the case, Neuroradiologists decided to carry out a bilateral arteriography of the head. This test also proved the existence of a direct posttraumatic CCF. In addition, this test also showed that the fistula presented high blood flow, with significant impact on intracranial hemodynamics (Fig. 1). Against this backdrop, neuroradiologists decided to treat the patient with endovascular approach. More in detail, they carried out a right cavernous sinus embolization with arterial access. In fact, through Seldinger technique (femoral approach) they performed the embolization of the lesion using 12 endovascular coils. In this sense, we would like to stress that the surgery was highly successful (Figs. 2,3). In fact, chemosis and pulsating exophthalmos of the right eye completely disappeared after surgery. Moreover, the patient referred improvement of visual acuity. Importantly, we also want to emphasise that the patient remained asymptomatic after 9 years of clinical monitoring.

## Discussion

Barrow’s classification of CCF is based on the pattern of arterial supply (6). Barrow type A (direct) usually present a single connection between the internal carotid artery and the ipsilateral cavernous sinus. This type of fistula usually show high flow and it is frequently caused by facial trauma (7). In contrast, indirect CCF are divided into 3 types: B, C and D. In this context, Barrow type B usually shows a connection between meningial branches of the internal carotid and the sinus. Barrow type C is characterized by connections between meningial branches of the external carotid and the sinus. Finally, Barrow type D is characterized by connections between meningial branches of the internal and external carotid and the sinus. Contrary to what has been stressed before about direct fistulas, indirect fistulas are low flow dural fistulas. This is an important assertion. In fact, in several cases, indirect fistulas may disappear spontaneously (6). Specifically, this type of fistulas could not present clinical manifestations; whereas, direct fistulas usually provoke multiple ocular complications (8). In this line, we want to emphasize that the ocular complications of carotid-cavernous fistulas are extremely varied. In fact, the list of possible ocular complications includes: pulsating exophthalmos, conjunctival chemosis, orbital blow, diplopia and decrease of vision (9). Specifically, the severity of these symptoms depends on the venous drainage capacity and on the intensity of flow.

Additionally, with respect to the diagnosis, we want to stress that angiographic study is essential for a proper management of these cases. In fact this test allows a safe diagnosis of carotid-cavernous fistulas. In view of that, cerebral angiography is considered the gold standard for diagnosing these pathologies.

Notably, the treatment of carotid-cavernous fistulas has undergone a major evolution over the last years. In fact, the performance of endovascular procedures has radically changed the natural evolution of this diseases. In this sense, we want to highlight that several reports proved the effectiveness of endovascular approach. In fact, according with numerous papers, transarterial embolization using intravascular balloons appears to be as the most effective procedure for treating direct carotid-cavernous fistulas (10,11). On the other hand, transvenous embolization represent the gold standar for treating indirect carotid-cavernous fistulas (if treatment is needed).

With these ideas in mind, it seems important to underline that the diagnosis and treatment of carotid-cavernous fistulas is not a simple process. From our point of view, the effective management of each case requires concerted efforts. In this sense, we underline that the Interventional Neuroradiology and Maxillofacial services worked together to achieve an accurate diagnosis.

Concluding, this report contains four points that are central to us: firstly, contrary to popular belief, our patient presented a progressive evolution of oculars complications. Consequently, the patient not required urgent medical attention. Secondly, angiographic study is essential for the proper management of these diseases. Thirdly, an adequate multidisciplinary approach is imperative to ensure a proper treatment. Interventional Neuroradiology and Maxillofacial services must work together to perform a correct diagnosis and treatment of the case. Finally, we would like to stress that our patient was treated with transarterial embolization using intravascular coils. Interestingly, although transarterial embolization with intravascular balloons is described as the most effective technique for treating direct carotid-cavernous fistulas, we decided to treat our patient using intravascular coils due to the greater experience of Interventional Neuroradiology service with this technique. In this line, we are pleased to assert that the patient remained asymptomatic after 9 years of clinical monitoring.

## Conclusions

Angiographic study is essential for the proper management of these diseases. Besides this, an adequate multidisciplinary support is imperative to ensure the proper diagnosis and treatment of CFF. In addition, we want to emphasize that the patients who suffered severe facial traumas require a long follow-up. In fact, certain complications of trauma might appear long time after the accident.
